# Synthesis and Evaluation of *in Vitro* Biological Activity of 4-Substituted Arylpiperazine Derivatives of 1,7,8,9-Tetrachloro-10,10-dimethoxy-4-azatricyclo[5.2.1.0^2,6^]dec-8-ene-3,5-dione

**DOI:** 10.3390/molecules14125189

**Published:** 2009-12-11

**Authors:** Jerzy Kossakowski, Magdalena Pakosinska-Parys, Marta Struga, Izabela Dybala, Anna E. Koziol, Paolo La Colla, Laura Ester Marongiu, Cristina Ibba, David Collu, Roberta Loddo

**Affiliations:** 1 Department of Medical Chemistry, Medical University, 3 Oczki Str., 02-007 Warsaw, Poland; 2 Faculty of Chemistry, Maria Curie-Sklodowska University, 20-031 Lublin, Poland; 3 Department of Biomedical Science and Technology, University of Cagliari, Cittadella Universitaria, 09042, Monserrato, Cagliari, Italy

**Keywords:** 1,7,8,9-tetrachloro-10,10-dimethoxy-4-azatricyclo[5.2.1.0^2,6^]dec-8-ene-3,5-dione, arylpiperazine derivatives, X-ray crystal structure analysis, antiviral activity

## Abstract

A series of twenty arylpiperazine derivatives of 1,7,8,9-tetrachloro-10,10-dimethoxy-4-azatricyclo[5.2.1.0^2,6^]dec-8-ene-3,5-dione have been prepared. These derivatives were tested *in vitro* with the aim of identifying novel lead compounds active against emergent and re-emergent human and cattle infectious diseases (AIDS, hepatitis B and C, tuberculosis, bovine viral diarrhea). In particular, these compounds were evaluated *in vitro* against representatives of different virus classes, such as a HIV-1 (Retrovirus), a HBV (Hepadnavirus) and the single-stranded RNA^+^ viruses Yellow fever virus (YFV) and Bovine viral diarrhea virus (BVDV), both belonging to the Flaviridae. Compounds **2c**, **2g** and **3d** showed a modest activity against CVB-2. The molecular structures of the starting imide **1** and one of propyl-piperazine derivatives, **3b**, have been determined by an X-ray crystallography study.

## Introduction

It is widely recognized that the utility of most antiviral agent is limited by the inherent toxicity of the compounds and the appearance of drug resistant virus following their continued use. As such, there is great interest in the identification of novel antiviral agents and lead compounds from which new antivirals might be synthesized. Viruses belonging to the Flaviviridae family have a single-stranded, positive-sense RNA genome (ssRNA^+^) [1]. Members of the Flaviviridae family can be classified into three genera: Hepaciviruses, Flaviviruses and Pestiviruses [2,3,4].

Some members of the Flaviviridae family can infect only animals, causing severe diseases usually followed by death [eg. bovine viral diarrhea virus (BVDV), classical swine fever virus (CSFV) and border disease virus (BDV)] [5]. In humans infections with Flaviviridae viruses may cause fulminant, hemorrhagic diseases [Yellow fever virus (YFV), dengue fever virus (DENV) and Omsk hemorrhagic fever virus (OHFV)], viral encephalitis [Japanese encephalitis virus (JEV), tick-borne encephalitis virus (TBEV), West Nile virus (WNV), St. Luis encephatitis virus (SLEV)] or hepatitis, formerly referred to as non-A, non-B hepatitis [hepatitis C virus (HCV)] [4,5,6]. Globally HCV is a major cause of human hepatitis [7,8]. Most infections become persistent, and about 60% of cases progress toward chronic liver disease, which in turn, can lead to development of cirrhosis, hepatocellular carcinoma, and liver failure [9,10]. There is a vaccine against the various Flaviviridae members with the exception of YFV; new therapies and preventative agents are strongly needed. 

Other important ssRNA^+^ viruses are those belonging to the Picornaviridae family. This family includes nine genera, some of which comprise major human pathogens, namely, Enterovirus (including Poliovirus, Coxsackievirus, Echovirus), Rhinovirus (approximately 105 serotypes), and Hepatovirus [Hepatitis A virus (HAV)]. The range of clinical syndromes associated with enteroviruses is legion and includes acute paralysis, aseptic meningitis, encephalitis, carditis, pleurodynia, conjunctivitis, hand food and mouth disease and other enanthems [11]. While many of these diseases are relatively benign, others, such as encephalitis and carditis, may be fatal. A fascinating aspect of these clinical syndromes is that different enteroviruses may be associated with the same clinical syndrome. Conversely, the same enterovirus may cause different clinical syndromes. Moreover, these associations are not always predictable: A given virus may behave differently, both clinically and epidemiologically, in different places at different times [12]. At present, no specific antiviral therapy is available for the treatment of Picornaviridae infections. 

Some arylpiperazine derivatives possess antienteroviral activity [13,14]. Piperazine fragment there are in anti-HIV agents bis(heteroaryl)piperazines (BHAPs), such as delavirdine and atevirdine [15]. Another arylpiperazine, vicriviroc, is currently under II phase of clinical investigation [16]. Inspired by these reports, we have recently synthesized and tested *in vitro* a series of arylpiperazine derivatives of a tricyclic imide for cytotoxicity and antiviral activity against representative viruses assays. The title compounds were also tested against representatives of other virus families. AZT (3’-azidothymidine), NM 108 (2’-β-methylguanosine), NM 176 (2’-ethynyl-D-cytidine), M 5255 (mycophenolic acid) and ACG (acycloguanosine) were used as reference inhibitors of ssRNA+, ssRNA- and DNA viruses, respectively.

## Results and Discussion

**Scheme 1:**
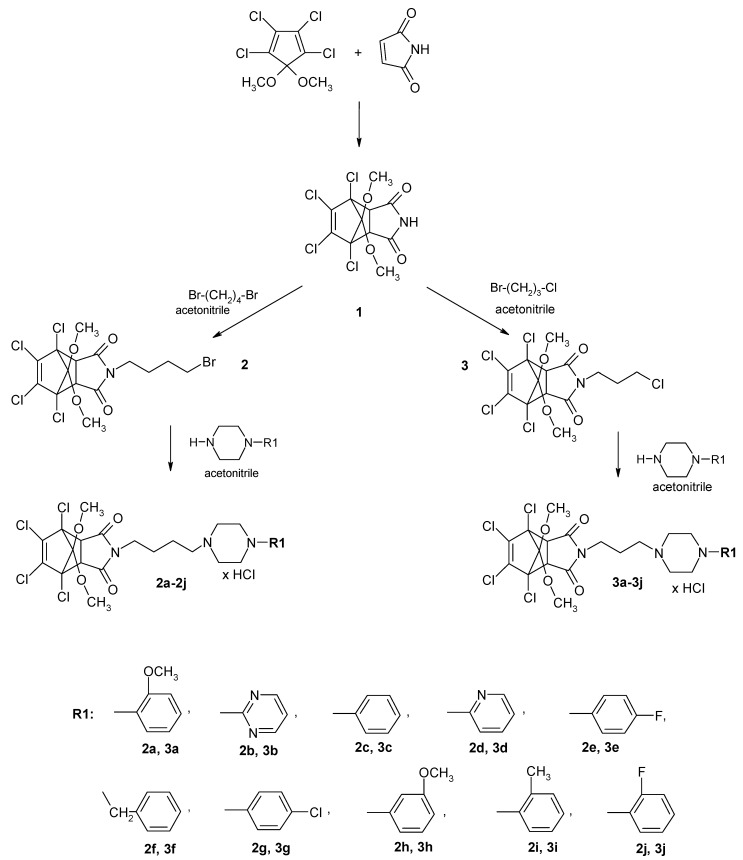
Compound Synthesis.

The preparation of the compounds was accomplished according to the reactions described in Scheme 1. The starting compound, 1,7,8,9-tetrachloro-10,10-dimethoxy-4-azatricyclo [5.2.1.0^2,6^]dec-8-ene-3,5-dione (**1**), was synthesized in a Diels-Alder reaction of 5,5-dimetoxy-1,2,3,4-tetracyclo-pentadiene with 1*H*-pyrrole-2,5-dione. 1,7,8,9-Tetrachloro-4-(4-chlorobutyl)- (**2**) and 1,7,8,9-tetrachloro-4-(3-chloropropyl)-10,10-dimethoxy-4-aza-tricyclo[5.2.1.0^2,6^]dec-8-ene-3,5-dione (**3**) have been prepared by the alkylation of imide **1** with 1,4-dibromobutane and 1-bromo-3-chloropropane, respectively. The alkylated derivatives were further substituted in acetonitrile with 4-aryl- and 4-heteroaryl piperazines in the presence of potassium carbonate and potassium iodide. For biological assays, the obtained arylpiperazine derivatives were converted into the corresponding hydrochloride salts in anhydrous methanol saturated with gaseous HCl to yield compounds **2a**–**2j** and **3a**–**3j** (Scheme 1). The new compounds were identified and their structure has been proven by ^1^H-NMR and elemental analyses (Table 1). 

**Figure 1:**
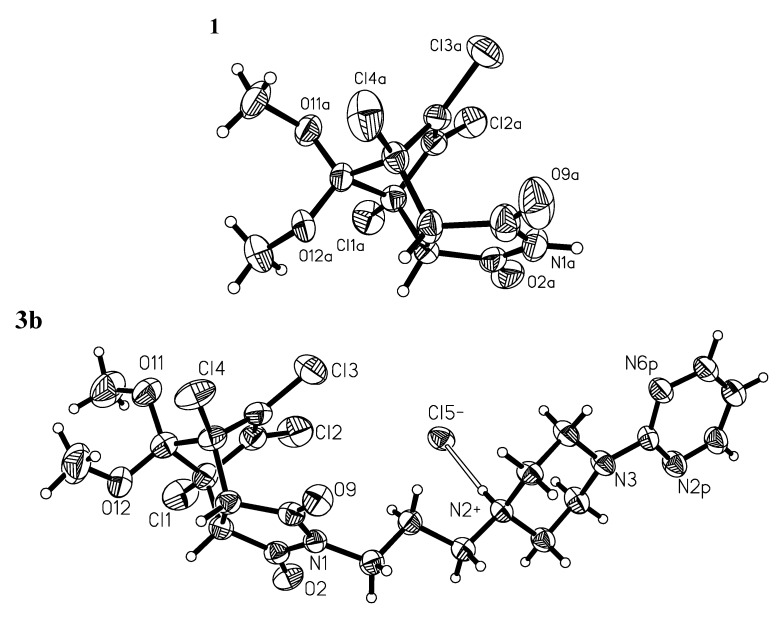
Molecular structures of compounds 1 and 3b.

The molecular structure of starting imide **1** and one of propylpiperazine derivatives, **3b**, has also been determined by an X-ray crystallography study (Table 2). The configuration of the cycloalkene skeleton of the imide **1** is *endo*, *cis* (Figure 1). This compound crystallizes with two symmetrically independent molecules, A and B, both adopting similar orientations of the –OCH_3_ groups in the solid state. The alternating molecules A and B are linked through four intermolecular N-H...O hydrogen bonds forming centrosymmetric tetramer. This type of molecular association excludes imide groups from further hydrophilic contacts.

The *N*-propyl-piperazine derivative **3b** adopts an extended conformation. In its protonated form it exists as a cation/anion pair (Figure 1); the observed geometry of the N^+^-H...Cl^–^ hydrogen bond is: N^+^...Cl^–^ distance of 3.032(6) Å and ∠N^+^-H...Cl^–^ angle of 175°. 

The synthesized arylpiperazine derivatives were evaluated *in vitro* in parallel cell-based assays for cytotoxicity and antiviral activity (Table 3) against viruses representative of two of the three genera of the Flaviviridae family, *i.e.*, Flaviviruses (YFV) and Pestiviruses (BVDV). The title compounds were also tested against representatives of other virus families. Among ssRNA+ were a Retrovirus (Human Immunodeficiency Virus type 1, HIV-1), two Picornaviruses (Coxsackie Virus type B2, CVB-2, and Poliovirus type-1, Sabin strain, Sb-1); among ssRNA- were a Paramyxoviridae (a Rhabdoviridae, Vesicular Stomatitis Virus, VSV) representative. Among double-stranded RNA (dsRNA) viruses was a Reoviridae representative (Respiratory Enteric Orphan Virus type-1, Reo-1). Two representatives of DNA virus families were also included: Herpes Symplex type 1, HSV-1 (Herpesviridae) and Vaccinia Virus, VV (Poxviridae). Although compound **1** was characterized by high cytotoxicity (Table 3), its derivatives possessed considerably low cytotoxicity. None of the compounds showed any activities against all viruses tested, with the exception of compounds **2c**, **2g** and **3d**, that showed a modest activity against CVB-2-mediated diseases and potentiality as therapeutic targets (EC_50_ range = 10–17 μM) (Table 3).

## Experimental

### General

Melting points were determined in a Kofler’s apparatus and are uncorrected. The ^1^H NMR spectra were recorded on a Bruker AVANCE DMX 400 spectrometer, operating at 400 MHz. The chemical shift values are expressed in ppm relative to TMS as an internal standard. Elemental analyses were recorded with a CHN 2400 Perkin-Elmer model. Flash chromatography was performed on Merck silica gel 60 (200–400 mesh) using chloroform/methanol (19:1 vol.) mixture as eluent. Analytical TLC was carried out on silica gel F_254_ (Merck) plates (0.25 mm thickness). 

*1,7,8,9-Tetrachloro-10,10-dimethoxy-4-azatricyclo[5.2.1.0^2,6^]dec-8-ene-3,5-dione* (**1**) was synthesized as previously described [17]. 

*1,7,8,9-Tetrachloro-4-(4-bromo-butyl)-10,10-dimethoxy-4-aza-tricyclo[5.2.1.0^2,6^]dec-8-ene-3,5-dione* (**2**). A mixture of imide **1** (0.5 g, 0.00138 mol), dibromobutane (0.9 g, 0.0027 mol) and anhydrous K_2_CO_3_ (0.5 g, 0.0036 mol) in acetonitrile (50 mL) was refluxed for 8 h. The inorganic precipitate was filtered off and the solvent was evaporated. For characterization data see Table 1.

*1,7,8,9-Tetrachloro-4-(3-chloro-propyl)-10,10-dimethoxy-4-aza-tricyclo[5.2.1.0^2,6^]dec-8-ene-3,5-dione* (**3**). A mixture of imide **1** (0.5 g, 0.00138 mol), 1-bromo-3-chloropropane (0.6 g, 0.00415 mol) and anhydrous K_2_CO_3_ (0.5 g, 0.0036 mol) in acetonitrile (50 mL) was refluxed for 8 h. The inorganic precipitate was filtered off, the solvent was evaporated. For characterization data see Table 1.

### General procedure for the preparation of arylpiperazine derivatives **2a**–**2j** and **3a**–**3j**

A mixture of compound **2** (0.3 g; 0.0006 mole) or **3** (0.3 g; 0.0007 mole), an appropriate amine (0.00137 or 0.0012 mole), anhydrous K_2_CO_3_ (0.3 g, 0.0022 mole) and KI (0.2 g, 0.0012 mole) was dissolved in acetonitrile (50 mL) and refluxed for 30 h. The solvent was evaporated, then the residue was purified by column chromatography (eluent: CH_2_Cl_2_-CH_3_OH, 95:5) to give compounds **2a**–**2j** and **3a**–**3j**, respectively.

### General procedure for the preparation of hydrochloride salts of compounds **2a–2j** and **3a–3j**

The solid product was dissolved in methanol saturated with gaseous HCl. The hydrochloride was precipitated by addition of diethyl ether. The crude product was crystallized from methanol/ethyl ether. Characterization data is given in Table 1.

### X-Ray crystal structure analysis of **1** and **3b** diethyl ether hemisolvate

Measurements were carried out at 292 K with a KM4 diffractometer using graphite monochromated CuK_α_ radiation (λ = 1.54178 Å) and ω/2θ scan mode. Intensities were corrected for absorption. Structure was solved by the SHELXS-97 program and refined by full-matrix least-squares on *F*^2^ using the SHELXL-97 program [18]. The non-H atoms were refined anisotropically. The H-atom positions were calculated from geometry and the ‘riding’ model for the C–H bonds was used in the refinement. The N-bonded H-atoms were located in the difference maps. Crystal data are listed in Table 2. The experimental details and final atomic parameters for **1** and **3b** have been deposited with the Cambridge Crystallographic Data Centre as supplementary material. Copies of the data can be obtained free of charge on application to The Director, CCDC, 12 Union Road, Cambridge CB2 1EZ, UK, (Fax: +44-1223-336-033; E-Mail: deposit@ccdc.cam.ac.uk or www: http://www.ccdc.cam.ac.uk).

### Cell-based assays

Compounds were dissolved in DMSO at 100 mM and then diluted in culture medium.

Cell lines were purchased from American Type Culture Collection (ATCC). The absence of mycoplasma contamination was checked periodically by the Hoechst staining method. Cell lines supporting the multiplication of RNA viruses were the following: CD4^+^ human T-cells containing an integrated HTLV-1 genome (MT-4); Madin Darby Bovine Kidney (MDBK); Baby Hamster Kidney (BHK-21) and Monkey kidney (Vero 76) cells.

#### Cytotoxicity Assays

For cytotoxicity tests, run in parallel with antiviral assays, MDBK, BHK and Vero 76 cells were resuspended in 96 multiwell plates at an initial density of 6 × 10^5^, 1 × 10^6^ and 5 × 10^5^ cells/mL, respectively, in maintenance medium, without or with serial dilutions of test compounds. Cell viability was determined after 48–120 hrs at 37 °C in a humidified CO_2_ (5%) atmosphere by the MTT method. The cell number of Vero 76 monolayers was determined by staining with the crystal violet dye.

For cytotoxicity evaluations, exponentially growing cells derived from human haematological tumors [CD4^+^ human T-cells containing an integrated HTLV-1 genome (MT-4)] were seeded at an initial density of 1 × 10^5^ cells/mL in 96 well plates in RPMI-1640 medium, supplemented with 10% fetal calf serum (FCS), 100 units/mL penicillin G and 100 µg/mL streptomycin. Cell cultures were then incubated at 37 °C in a humidified, 5% CO_2_ atmosphere in the absence or presence of serial dilutions of test compounds. Cell viability was determined after 96 hrs at 37 °C by the 3-(4,5-dimethylthiazol-2-yl)-2,5-diphenyl-tetrazolium bromide (MTT) method [18].

#### Antiviral assays

Activity of compounds against Human Immunodeficiency virus type-1 (HIV-1) was based on inhibition of virus-induced cytopathogenicity in MT-4 cells acutely infected with a multiplicity of infection (m.o.i.) of 0.01. Briefly, RPMI (50 μL) containing 1 × 10^4^ MT-4 were added to each well of flat-bottom microtitre trays containing RPMI (50 μL), without or with serial dilutions of test compounds. Then, an HIV-1 suspension (20 μL) containing 100 CCID_50_ were added. After a 4-day incubation, cell viability was determined by the MTT method.

Activity of compounds against Yellow fever virus (YFV) and Reo virus type-1 (Reo-1) was based on inhibition of virus-induced cytopathogenicity in acutely infected BHK-21 cells. Activities against Bovine viral diarrhoea virus (BVDV), in infected MDBK cells, were also based on inhibition of virus-induced cytopathogenicity.

BHK and MDBK cells were seeded in 96-well plates at a density of 5 × 10^4^ and 3 × 10^4^ cells/well, respectively, and were allowed to form confluent monolayers by incubating overnight in growth medium at 37 °C in a humidified CO_2_ (5%) atmosphere. Cell monolayers were then infected with 50 µL of a proper virus dilution (in serum-free medium) to give an m.o.i = 0.01. One hr later, MEM Earle’s medium (50 µL), supplemented with inactivated foetal calf serum (FCS), 1% final concentration, without or with serial dilutions of test compounds, were added. After 3-4 days incubation at 37 °C, cell viability was determined by the MTT method.

Activity of compounds against Coxsackie virus, B-2 strain (CVB-2), Polio virus type-1 (Polio-1), Sabin strain, Vesicular Stomatitis Virus (VSV), Vaccinia Virus (VV) and Herpes Virus 1 (HSV-1), in infected Vero 76 cells, was determined by plaque reduction assays in Vero 76 cell monolayers. To this end, Vero 76 cells were seeded in 24-well plates at a density of 2 × 10^5^ cells/well and were allowed to form confluent monolayers by incubating overnight in growth medium at 37 °C in a humidified CO_2_ (5%) atmosphere. Then, monolayers were infected with appropriate virus dilutions (250 µL) to give 50−100 PFU/well. Following removal of unadsorbed virus, Dulbecco’s modified Eagle’s medium (500 µL), supplemented with 1% inactivated FCS and 0.75% methyl cellulose, without or with serial dilutions of test compounds, were added. Cultures were incubated at 37 °C for 2 (Sb-1 and VSV) or 3 (CVB-2, VV and HSV-1) and then fixed with PBS containing 50% ethanol and 0.8% crystal violet, washed and air-dried. Plaques were then counted. 50% effective concentrations (EC_50_) were calculated by linear regression technique.

AZT (3’-azido-thymidine), NM 108 (2’-ß-methyl-guanosine), NM 176 (2’-ethynyl-D-citidine), M 5255 (Mycophenolic Acid) and ACG (acycloGuanosine) were used as reference inhibitors of ssRNA+, ssRNA- and DNA viruses, respectively.

## Conclusions

A series of twenty arylpiperazine derivatives of 1,7,8,9-tetrachloro-10,10-dimethoxy-4-azatricyclo[5.2.1.0^2,6^]dec-8-ene-3,5-dione have been prepared. These derivatives were tested *in vitro* with the aim of identifying novel lead compounds active against emergent and re-emergent human and cattle viral infectious diseases. Compounds **2c**, **2g** and **3d** showed activity against CVB-2. Molecular structure of starting imide **1** and one of the propyl-piperazine derivatives, **3b**, have been determined by an X-ray crystallography study.

## Figures and Tables

**Table 1:** Physical and analytical data of compounds given in Scheme 1.

Comp.	M.p. (°C)	Yield (%)	Formula	Analysis Calculated/Found			^1^H-NMR, δ_H_ (*J* in Hz), solvent–CDCl_3_
				%C	%H	%N	
**2**	oil	72	C_14_H_14_Cl_5_N O_4_	36.32 36.23	3.25 3.58	2.82 3.09	3.64 (s, 3H, OCH_3_); 3.62 (s, 3H, OCH_3_); 3.44–3.37 (m, 6H, CH, CH_2_); 2.03–2.0 (m, 2H, CH_2_); 1.87–1.81 (m, 1H, CH_2_); 1.67–1.57 (m, 1H, CH_2_)
**3**	oil	74	C_15_H_16_BrCl_4_NO_4_	38.43 38.39	3.23 3.34	3.20 3.21	3.65 (s, 3H, OCH_3_); 3.63 (s, 2H, CH); 3.59 (s, 3H, OCH_3_); 3.60–3.59 (m, 2H, CH_2_); 3.53–3.50 (t, 2H, CH_2_, *J* = 6.4); 1.97–1.91 (t, 2H, CH_2_, *J* = 10.2)
**2a**	233	85	C_26_H_32_Cl_5_N_3_O_5_	48.50 48.37	5.01 5.25	6.53 6.34	13.3 (s, 1H, HCl); 8.17–8.15 (d, 1H, CH_arom._, *J* = 8); 7.48–7.44 (t, 1H, CH_arom._, *J* = 8.6); 7.09–7.03 (m, 2H, CH_arom._); 5.03–4.98 (m, 2H, CH_2_N); 4.35–4.33 (m, 2H, CH_2_N); 4.06 (s, 3H, OCH_3_); 3.73 (s, 2H, CH); 3.65 (s, 3H, OCH_3_); 3.58 (s, 3H, OCH_3_); 3.65–3.56 (m, 2H, CH_2_N); 3.23–3.2 (m, 4H, CH_2_N); 1.96–1.92 (m, 2H, CH_2_); 1.65–1.61 (m, 2H, CH_2_)
**2b**	256	86	C_23_H_28_Cl_5_N_5_O_4_	44.86 44.67	4.58 4.87	11.37 11.13	13.04 (s, 1H, HCl); 8.36 (s, 2H, CH_arom._); 6.64 (s, 1H, CH_arom._); 4.89–4.85 (m, 2H, CH_2_N); 3.87–3.91 (m, 2H, CH_2_N); 3.70 (s, 2H, CH); 3.65 (s, 3H, OCH_3_); 3.58 (s, 3H, OCH_3_); 3.46–3.43 (m, 2H, CH_2_N); 3.0–2.99 (m, 2H, CH_2_N); 2.79–2.77 (m, 2H,CH_2_N); 1.96–1.92 (m, 2H, CH_2_); 1.60–1.58 (m, 2H, CH_2_)
**2c**	243	88	C_25_H_30_Cl_5_N_3_O_4_	48.92 48.79	4.93 5.0	6.85 6.81	12.78 (s, 1H, HCl); 7.34–7.30 (s, 2H, CH_arom._); 7.02–7.04 (s, 3H, CH_arom._); 3.76 (s, 2H, CH); 3.65 (s, 3H, OCH_3_); 3.58 (s, 3H, OCH_3_); 3.65–3.56 (m, 6H, CH_2_N); 3.46–3.44 (m, 2H, CH_2_N); 3.07–2.0 (m, 2H,CH_2_N); 1.97–1.95 (m, 2H, CH_2_); 1.63–1.60 (m, 2H, CH_2_)
**2d**	248	76	C_24_H_29_Cl_5_N_4_O_4_	46.89 46.78	4.75 4.95	9.11 9.02	13.01 (s, 1H, HCl); 8.01 (s, 2H, CH_arom._); 7.13–7.06 (s, 1H, CH_arom._); 4.67–4.60 (m, 2H, CH_2_N); 4.21–4.19 (m, 2H, CH_2_N); 3.73 (m, 2H, CH_2_N); 3.65 (s, 2H, CH); 3.65 (s, 3H, OCH_3_); 3.57 (s, 3H, OCH_3_); 3.27–3.15 (m, 4H, CH_2_N); 1.94–1.92 (m, 2H, CH_2_); 1.63–1.61 (m, 2H, CH_2_)
**2e**	206	79	C_25_H_29_Cl_5_FN_3_O_4_	47.53 47.25	4.63 4.64	6.65 6.51	13.25 (s, 1H, HCl); 7.61–7.6 (m, 2H, CH_arom._); 7.15–7.12 (m, 2H, CH_arom._); 4.39–4.35 (m, 2H, CH_2_N); 3.85–3.83 (m, 2H, CH_2_N); 3.72 (s, 2H, CH); 3.65 (s, 3H, OCH_3_); 3.58 (s, 3H, OCH_3_); 3.65–3.56 (m, 4H, CH_2_N); 3.49–3.41 (m, 2H, CH_2_N); 1.95–1.92 (m, 2H, CH_2_); 1.65–1.63 (m, 2H, CH_2_).
**2f**	211	68	C_26_H_32_Cl_5_N_3_O_4_	49.74 49.56	5.14 5.41	6.69 6.34	12.96 (s, 1H, HCl); 7.66–7.63 (m, 2H, CH_arom._); 7.48–7.45 (m, 3H, CH_arom._); 4.26–4.24 (m, 2H, CH_2_); 4.02–4.0 (m, 4H, CH_2_N); 3.72 (s, 2H, CH); 3.64 (s, 3H, OCH_3_); 3.58 (s, 3H, OCH_3_); 3.46–3.41 (m, 4H, CH_2_N); 3.16–3.14 (m, 2H, CH_2_N); 1.89–1.85 (m, 2H, CH_2_); 1.69–1.62 (m, 2H, CH_2_)
**2g**	230	87	C_25_H_29_Cl_6_N_3_O_4_	46.32 46.26	4.51 4.81	6.48 6.68	13.23 (s, 1H, HCl); 7.58–7.56 (m, 2H, CH_arom._); 7.44–7.42 (s, 2H, CH_arom._); 4.44–4.38 (m, 2H, CH_2_N); 3.85–3.83 (m, 2H, CH_2_N); 3.72 (s, 2H, CH); 3.65 (s, 3H, OCH_3_); 3.58 (s, 3H, OCH_3_); 3.65–3.56 (m, 2H, CH_2_N); 3.50–3.47 (m, 2H, CH_2_N); 3.19–3.17 (m, 2H, CH_2_N); 1.95–1.92 (m, 2H, CH_2_); 1.65–1.63 (m, 2H, CH_2_)
**2h**	231	86	C_26_H_32_Cl_5_N_3_O_5_	48.5 48.42	5.01 5.23	6.53 6.56	12.91 (s, 1H, HCl); 7.25–7.23 (m, 1H, CH_arom._); 6.72–6.63 (s, 3H, CH_arom._); 3.98–3.92 (m, 2H, CH_2_N); 3.80 (s, 3H, OCH_3_); 3.76 (s, 2H, CH); 3.65 (s, 3H, OCH_3_); 3.58 (s, 3H, OCH_3_); 3.65–3.56 (m, 4H, CH_2_N); 3.3–3.28 (m, 2H, CH_2_N); 1.96–1.92 (m, 2H, CH_2_); 1.63–1.6 (m, 2H, CH_2_)
**2i**	213	84	C_26_H_32_Cl_5_N_3_O_4_	49.74 49.72	5.14 5.18	6.69 6.44	12.97 (s, 1H, HCl); 7.71–7.7 (m, 1H, CH_arom._); 7.36–7.3 (s, 3H, CH_arom._); 4.68–4.46 (m, 4H, CH_2_N); 3.83 (s, 2H, CH); 3.74–3.65 (m, 2H. CH_2_N); 3.65 (s, 3H, OCH_3_); 3.58 (s, 3H, OCH_3_); 3.50–3.48 (m, 2H, CH_2_N); 3.24–3.22 (m, 2H, CH_2_N); 2.74 (s, 3H, CH_3_); 1.96–1.924 (m, 2H, CH_2_); 1.66–1.63 (m, 2H, CH_2_)
**2j**	215	76	C_25_H_29_Cl_5_FN_3_O_4_	47.53 47.49	4.63 4.78	6.65 6.55	12.72 (s, 1H, HCl); 7.1–6.96 (m, 4H, CH_arom._); 4.77–4.74 (m, 2H, CH_2_N); 3.76 (s, 2H, CH); 3.72–3.65 (m, 2H, CH_2_N); 3.65 (s, 3H, OCH_3_); 3.58 (s, 3H, OCH_3_); 3.5–3.4 (m, 2H, CH_2_N); 3.06–3.05 (m, 2H, CH_2_N); 1.97–1.96 (m, 2H, CH_2_); 1.63–1.61 (m, 2H, CH_2_)
**3a**	185	87	C_25_H_30_Cl_5_N_3_O_5_	50.61 50.60	4.93 4.91	7.08 7.09	7.02–6.99 (m, 1H, CH_arom._); 6.93–6.91 (s, 2H, CH_arom._); 6.89–6.85 (m, 1H, CH_arom._); 3.86 (s, 3H, OCH_3_); 3.64 (s, 5H, CH, OCH_3_); 3.59 (s, 3H, OCH_3_); 3.54–3.52 (m, 2H, CH_2_N); 3.16–3.14 (m, 4H, CH_2_N); 2.74–2.7 (m, 4H, CH_2_N); 2.54–2.52–1.92 (m, 2H, CH_2_N); 1.78–1.76 (m, 2H, CH_2_)
**3b**	244	78	C_22_H_26_Cl_5_N_5_O_4_	46.74 46.52	4.46 4.61	12.39 12.28	11.17 (s, 1H, HCl); 8.44–8.43 (d, 2H, CH_arom._, *J* = 4.8); 6.77–6.74 (t, 1H, CH_arom._, *J* = 4.6); 3.81 (m, 2H, CH_2_N); 3.60 (s, 2H, CH); 3.49–3.47 (m, 6H, CH_2_N); 3.41 (s, 6H, OCH_3_); 3.06–3.2.97 (m, 4H, CH_2_N); 1.87–1.83 (m, 2H, CH_2_)
**3c**	246	75	C_24_H_28_Cl_5_N_3_O_4_	48.06 48.03	4.71 4.59	7.01 6.8	12.89 (s, 1H, HCl); 7.33–7.29 (m, 2H, CH_arom._); 7.01–6.99 (m, 3H, CH_arom.,_); 3.74–3.72 (m, 6H, CH_2_N); 3.65 (s, 8H, CH, OCH_3_); 3.59–3.54 (m, 2H, CH_2_N); 3.5–2.45 (m, 4H, CH_2_N); 2.18–2.16 (m, 2H, CH_2_)
**3d**	248	78	C_23_H_27_Cl_5_N_4_O_4_	45.98 45.58	4.53 4.94	9.33 9.02	13.04 (s, 1H, HCl); 8.21 (d, 1H, CH_arom.,_ *J* = 4); 7.6–7.58 (m, 2H, CH_arom._); 6.8–6.77 (s, 2H, CH_arom._); 4.34 (m, 2H, CH_2_N); 3.90–3.89 (m, 2H, CH_2_N); 3.8 (s, 2H CH); 3.65 (s, 6H, OCH_3_); 3.63–3.37 (m, 4H, CH_2_N);3.0–2.86 (m, 4H, CH_2_N); 2.17–2.16 (m, 2H, CH_2_)
**3e**	237	80	C_24_H_27_Cl_5_FN_3_O_4_	46.66 46.22	4.41 4.73	6.80 6.51	12.83 (s, 1H, HCl); 7.09–7.03 (m, 4H, CH_arom._); 3.76–3.45 (m, 8H, CH_2_N); 3.65 (s, 5H, CH, OCH_3_); 3.58 (s, 3H, OCH_3_); 3.15–3.1 (m, 4H, CH_2_N); 2.18–2.14 (m, 2H, CH_2_)
**3f**	241	64	C_25_H_30_Cl_5_N_3_O_4_	48.92 48.72	4.93 4.85	6.85 6.72	7.64–6.61 (m, 2H, CH_arom._); 7.47–7.45 (s, 3H, CH_arom._); 4.23–4.21 (m, 2H, CH_2_); 3.69–3.67 (m, 4H, CH_2_N); 3.65–3.44 (m, 6H, CH_2_N); 3.65 (s, 5H, CH, OCH_3_); 3.57 (s, 3H, OCH_3_); 3.11–3.08 (m, 2H, CH_2_N); 2.03–2.0 (m, 2H, CH_2_)
**3g**	250	85	C_24_H_27_Cl_6_N_3_O_4_	45.45 45.36	4.29 4.35	6.63 6.46	13.13 (s, 1H, HCl); 7.33–7.26 (m, 4H, CH_arom._); 4.05–4.0 (m, 2H, CH_2_N); 3.74–3.71 (m, 2H, CH_2_N); 3.65 (s, 5H, CH, OCH_3_); 3.59 (s, 3H, OCH_3_); 3.65–3.58 (m, 4H, CH_2_N); 3.42–3.4 (m, 2H, CH_2_N); 3.14–3.12 (m, 2H, CH_2_N); 2.16–2.14 (m, 2H, CH_2_)
**3h**	219	81	C_25_H_30_Cl_5_N_3_O_5_	47.68 47.82	4.80 5.04	6.67 6.52	12.9 (s, 1H, HCl); 7.23–7.21 (m, 1H, CH_arom._); 6.68–6.59 (m, 3H, CH_arom._); 3.9 (s, 3H, OCH_3_); 3.89–3.74 (m, 4H, CH_2_N); 3.65 (s, 5H, CH, OCH_3_); 3.58 (s, 3H, OCH_3_); 3.64–3.57 (m, 4H, CH_2_N); 3.18–3.1 (m, 4H, CH_2_N); 2.17–2.14 (m, 2H, CH_2_).
**3i**	229	75	C_25_H_30_Cl_5_N_3_O_4_	48.92 48.82	4.93 5.13	6.85 6.75	12.83 (s, 1H, HCl); 7.16–7.12 (m, 1H, CH_arom._); 6.34–6.32 (m, 3H, CH_arom._); 3.93–4.0 (m, 2H, CH_2_N); 3.75–3.71 (m, 2H, CH_2_N); 3.65 (s, 5H, CH, OCH_3_); 3.59 (s, 3H, OCH_3_); 3.58–3.31 (m, 4H, CH_2_N); 3.15–3.12 (m, 2H, CH_2_N); 2.43–2.4 (m, 2H, CH_2_N); 2.17–2.14 (m, 2H, CH_2_)
**3j**	224	73	C_24_H_27_Cl_5_FN_3_O_4_	46.66 46.70	4.41 4.66	6.80 6.57	12.92 (s, 1H, HCl); 7.08–7.04 (m, 4H, CH_arom._); 3.74–3.72 (m, 2H, CH_2_N); 3.69–3.67 (m, 2H, CH_2_N); 3.65 (s, 5H, CH, OCH_3_); 3.58 (s, 3H, OCH_3_); 3.57–3.45 (m, 4H, CH_2_N); 3.08–3.04 (m, 4H, CH_2_N); 17–2.14 (m, 2H, CH_2_)

**Table 2:** Crystal data and refinement parameters for **1** and **3b**.

		1	3b
Empirical formula		C_11_H_9_Cl_4_NO_4_	C_22_H_26_Cl_5_N_5_O_4_ × (C_2_H_5_O)_0.5_
Formula weight		360.99	638.79
Crystal system, space group		triclinic, *P*-1	monoclinic, *P*2_1_/*c*
Unit cell dimensions	*a* (Å)	8.304(2)	16.230(3)
*b* (Å)		13.554(3)	8.642(2)
*c* (Å)		14.642(3)	22.153(4)
α (°)		111.97(3)	90
β (°)		96.28(3)	104.03(3)
γ (°)		103.63(3)	90
Volume (Å^3^)		1449.2(6)	3014.5(10)
Z, Calculated density (g cm^–3^)		4, 1.655	4, 1.408
F(000)		728	1324
Absorption coefficient (mm^-1^)		7.548	4.725
Crystal size (mm)		0.32 × 0.25 × 0.18	0.42 × 0.19 × 0.11
Absorption corr.; T min, max		0.054, 0.151	0.145, 0.245
θ range for data collection (°)		5.6–72.9	5.5–75.8
Limiting indices		-10 ≤ *h* ≤ 0, -15 ≤ *k* ≤ 15, -16 ≤ *l* ≤ 17	-20 ≤ *h* ≤ 19, 0 ≤ *k* ≤ 10, 0 ≤ *l* ≤ 27
Reflections collected / unique / observed [*I* > 2σ (*I*)]		5960 / 5596 / 2773 [*R*_int_ = 0.0424]	6295 / 6162 / 2021 [*R*_int_ = 0.0354]
Data / parameters		5596 / 366	6162 / 365
Goodness-of-fit on *F*^2^		1.074	0.944
Final R indices [*I* > 2σ (*I*)]		*R*_1_ = 0.0452, *wR*_2_ = 0.1370	*R*_1_ = 0.0620, *wR*_2_ = 0.1515
Max and min Δρ (e Å^-3^)		0.43 and -0.32	0.59 and -0.39
Extinction coeff.		0.0018(3)	0.0019(3)
CCDC No		713452	713453

**Table 3:** Cytotoxicity and antiviral activity of 4-substituted a arylpiperazine derivatives of 1,7,8,9-tetrachloro-10,10-dimethoxy-4-azatricyclo[5.2.1.0^2,6^]dec-8-ene-3,5-dione.

Compd.	^a^MT-4 CC_50_ [µM]	^b^HIV-1 EC_50_ [µM]	^c^MDBK CC_50_ [µM]	^d^BFDV EC_50_ [µM]	^e^BHK-21 CC_50_ [µM]	^f^YFV EC_50_ [µM]	^f^Reo-1 EC_50_ [µM]
**1**	>100	>100	-	**-**	-	-	-
**2a**	24	>24	86	**>**86	13	>13	>13
**2b**	>100	>100	>100	>100	>100	>100	>100
**2c**	45	>45	>100	>100	>100	>100	>100
**2d**	36	>36	>100	>100	>100	>100	>100
**2e**	16	>16	>100	>100	64	>64	>64
**2g**	11	>11	>100	>100	>100	>100	>100
**2h**	18	>18	>65	>65	11	>11	>11
**2i**	17	>17	>14	>14	7	>7	>7
**2j**	24	>24	>100	>100	30	>30	>30
**3a**	32	>32	73	>73	22	>22	>22
**3c**	30	>30	100	>100	29	>29	>29
**3d**	53	>53	>100	>100	>100	>100	>100
**3e**	34	>34	89	>89	36	>36	>36
**3i**	20	>20	24	>24	9	>9	>9
**Compd.**	**^g^Vero-76** **CC_50_ [µM]**	**^h^HSV-1** **EC_50_ [µM]**	**^h^VV** **EC_50_ [µM]**	**^h^VSV** **EC_50_ [µM]**	**^h^CVB-2** **EC_50_ [µM]**	**^h^SB-1** **EC_50_ [µM]**	
**1**	**-**	-	-	-	-	-	
**2a**	80	>80	>80	>80	>80	>80	
**2b**	95	>95	>95	>95	>95	>95	
**2c**	84	>84	>84	>84	**13**	>84	
**2d**	95	>95	>95	>95	>95	>95	
**2e**	60	>60	>60	>60	>60	>60	
**2g**	95	>95	>95	>95	**10**	>95	
**2h**	70	>70	>70	>70	>70	>70	
**2i**	45	>45	>45	>45	>45	>45	
**2j**	52	>52	>52	>52	>52	>52	
**3a**	60	>60	>60	>60	>60	>60	
**3c**	70	>70	>70	>70	>70	>70	
**3d**	82	>82	>82	>82	**17**	>82	
**3e**	75	>75	>75	>75	>75	>75	
**3i**	34	>34	>34	>34	>34	>34	

^a^ Compd. concn. (Mμ) required to reduce the viability of mock-infected MT-4 (CD4+ Human T-cells containing an integrated HTLV-1 genome) cells by 50%, as determined by the colorimetric MTT method; ^b^ Compound concentration (Mμ) required to achieve 50% protection of MT-4 cells from the HIV-1-induced cytopathogenicity, as determined by the MTT method; ^c^ Compd. concn. (Mμ) required to reduce the viability of mock-infected MDBK (Bovine normal kidney) cells by 50%, as determined by the MTT method; ^d^ Compd. concn. (Mμ) required to achieve 50% protection of MDBK cells from the BVDV (Bovine Viral Diarrhea Virus)-induced cytopathogenicity, as determined by the MTT method; ^e^ Compd. concn. (Mμ) required to reduce the viability of mock-infected BHK (Hamster normal kidney fibroblast) monolayers by 50%, as determined by the MTT method; ^f^ Compound concentration (Mμ) required to achieve 50% protection of BHK (Kidney fibroblast) cells from the YFV (Yellow Fever Virus) and Reo (Reovirus 1)-induced cytopathogenicity, as determined by the MTT method; ^g^ Compd. concn. (μM) required to reduce the viability of mock-infected VERO-76 (Monkey normal kidney) monolayers by 50%, as determined by the MTT method; ^h^ Compd. concn. (μM) required to reduce the plaque number of HSV-1 (Herpesvirus 1), VV (Vaccinia Virus), VSV (Vesicular Stomatitis Virus), CVB-2 (Coxsackievirus B2), Sb-1 (Poliovirus 1) and RSV (Respiratory Syncytial Virus) by 50% in VERO-76 monolayers
